# Apolipoprotein E-Mimetic Peptide COG1410 Enhances Retinal Ganglion Cell Survival by Attenuating Inflammation and Apoptosis Following TONI

**DOI:** 10.3389/fnins.2019.00980

**Published:** 2019-09-13

**Authors:** Li Kuai, Jianhua Peng, Yong Jiang, Zheng Zheng, Xiyuan Zhou

**Affiliations:** ^1^Department of Ophthalmology, The Second Affiliated Hospital of Chongqing Medical University, Chongqing, China; ^2^Department of Ophthalmology, The Affiliated Hospital of Southwest Medical University, Luzhou, China; ^3^Department of Neurosurgery, The Affiliated Hospital of Southwest Medical University, Luzhou, China; ^4^Neurosurgery Clinical Medical Research Center of Sichuan Province, Luzhou, China; ^5^Academician Expert Workstation, The Affiliated Hospital of Southwest Medical University, Luzhou, China; ^6^Laboratory of Neurological Diseases and Brain Functions, The Affiliated Hospital of Southwest Medical University, Luzhou, China

**Keywords:** traumatic optic nerve injury, inflammation, apoptosis, COG1410, magnetic resonance imaging

## Abstract

Vision loss after traumatic optic nerve injury (TONI) is considered irreversible because of the retrograde loss of retinal ganglion cells (RGCs), which undergo inflammation and apoptosis. Previous studies have shown that COG1410, a mimic peptide derived from the apolipoprotein E (apoE) receptor binding region, shows neuroprotective activity in acute brain injury. However, the detailed role and mechanisms of COG1410 in RGC survival and vision restoration after TONI are poorly understood. The current study aimed to investigate the effects of COG1410 on inflammation and apoptosis in a mouse model of TONI. The results showed that TONI-induced visual impairment was accompanied by optic nerve inflammation, apoptosis, edema, and RGC apoptosis. COG1410 significantly prevented the decrease in visual from ever occurring, attenuated inflammation and apoptosis, and reduced optic nerve edema and RGC apoptosis compared with vehicle treatment. These data identify protective roles of COG1410 in the inflammatory and apoptotic processes of TONI, as well as strategies for its treatment.

## Introduction

Traumatic optic nerve injury (TONI) is a leading cause of irreversible visual impairment worldwide and can even cause blindness (Crair and Mason, 2016). Retinal ganglion cells (RGCs), the projection neurons of the eye, cannot regenerate their axons once the optic nerve has been injured and soon begin to die (Li H.J. et al., 2018). Optic nerve (ON) injuries are frequently associated with degeneration of RGCs due to primary trauma to their axons that travel through the optic nerve to the brain (Xu et al., 2018). Currently, a large number of studies have focused on the promotion of neural regeneration, predominantly through the removal of inhibitory factors that prevent axonal regeneration following injury. However, medical and surgical treatments have all failed to improve outcomes, and some treatments carry significant risks (Steinsapir and Goldberg, 2011). After TONI, damaged RGCs die by apoptosis. Apoptotic cells initiate a highly controlled cascade of events, activation and proliferation of microglia (Bennett et al., 2016; Nadal-Nicolas et al., 2017), suggesting that microglia-mediated inflammatory injury may be an important cause of optic nerve injury.

Inflammation plays an important role in the process of secondary damage in the central nervous system (CNS). ON injury can cause local production of inflammation-related cytokines, which might complicate potential therapeutic strategies for RGC regeneration (Laha et al., 2017; Li et al., 2017). Mouse optic nerve is comprised by 500 + proteins and 200 + lipids, which react to injury in complex ways, involving inflammatory response and apoptosis, but also necrotic and regenerative processes (Tzekov et al., 2016). Collectively, inflammatory responses might contribute to RGC loss (Maes et al., 2017). Thus, a therapeutic strategy targeting inflammation would likely prevent neuronal damage and loss of vision following optic nerve trauma.

Apolipoprotein E (apoE) is the major apolipoprotein in the CNS and predisposes patients to a favorable outcome of neurological disorders (Jiang et al., 2006, 2007; Verghese et al., 2011; Peng et al., 2017). ApoE not only participates in lipid metabolism but also exerts anti-inflammatory and anti-apoptotic effects via binding to its functional receptors (Pocivavsek et al., 2009). Our recent study revealed that the apoE-mimetic peptide COG1410 shows neuroprotective effects, characterized by anti-inflammation and anti-apoptosis, in mouse models of traumatic brain injury (TBI) and hemorrhagic stroke (Wu et al., 2016; Qin et al., 2017). However, the detailed role and mechanisms of COG1410 in RGCs protection and vision restoration after TONI are poorly understood.

Thus, a deeper understanding of these opposing effects will be important for exploiting the modulation of inflammatory process to promote neural repair while minimizing bystander damage. In the present study, we investigate neuroprotective effect of COG1410 after TONI and to determine the possible underlying mechanisms of any observed effects.

## Materials and Methods

### Animals

A total of 110 wild-type (WT) C57BL/6J male mice, 10 to 12-weeks-old and weighing 18–23 g, were used in our experiments. The mice were labeled after removal from the feeding center and randomly divided into different groups (Sham group, TONI group, TONI + vehicle group, and TONI + COG1410 group) using the randomizer in Excel. In this study, all procedures were evaluated and approved by the Animal Care and Use Committee of Chongqing Medical University in Chongqing, China.

### Animal Model

Mice were weighed and anesthetized with pentobarbital sodium (50 mg/kg) by intraperitoneal injection, followed by routine disinfection and draping. A small incision was made in the superior and lateral conjunctiva (left), and forceps were gently used to expose the optic nerve. The left optic nerve was crushed for 10 s using self-closing forceps at a site approximately 1–2 mm posterior to the globe. After surgery, ophthalmic ointment (Daiichi-Sankyo, Shanghai, China) was applied to both eyes to ensure that the corneas did not dry out and to protect against infection. In the Sham group, all the operational procedures except the optic nerve crush were repeated. All models of optic nerve injury were constructed in the left eye.

### Drug Administration

The apoE-mimic peptide COG1410 was kindly provided by Prof. Michael P. Vitek at a purity of 95%. COG1410 was dissolved in a sterile 0.9% saline solution immediately prior to use, and the solution was injected into the tail vein at a dose of 1 mg/kg. The peptide was administered immediately after surgery, followed by a once-daily intravenous injection until the day before the mice were sacrificed. The vehicle groups underwent injections of only sterile 0.9% saline at each time-point. The investigator who administered the treatment was different from the investigator who performed the surgeries, and a single investigator who was blinded to the injury status and treatment regimens of the animals performed all assessments.

### Flash Visual Evoked Potentials (F-VEPs)

Prior to collection, mice were dark-adapted overnight, dilated with tropicamide for 10 min, and anesthetized with pentobarbital sodium (50 mg/kg) by intraperitoneal injection. Mice were placed on the heated surface of the Celeris system (Diagnosys LLC, Lowell, MA, United States) to maintain normal body temperature. Celeris corneal electrodes with integrated stimulators were placed on the lubricated corneas, and subdermal platinum electrodes were placed in the snout and back of the head at the location of the visual cortex. The left and right eyes were exposed independently to 50 flashes of 1 Hz, 0.5 cd.s/m^2^ white light.

### Magnetic Resonance Imaging (MRI) Scan

After flash visual evoked potential (F-VEP) test, magnetic resonance imaging (MRI) was performed using a 7.0 Tesla animal scanner (Bruker BioSpin, Ettlingen, Germany) as previously described (Cao et al., 2016). Briefly, mice were anesthetized using a gas mixture (induction, 5% isoflurane with 1 L/min O_2_; maintenance, 1% isoflurane with 1 L/min O_2_) and mounted in a Bruker animal bed. The body temperature was maintained at 37°C, and the respiratory rate was continuously monitored. T2-weighted images were acquired using RARE (repetition time = 4000 ms, echo time = 45 ms, RARE factor = 8, 0.5 mm, field of view = 2.5 cm, 256 × 256). Images were calculated with Bruker ParaVision 6.0 software (Bruker BioSpin, Ettlingen, Germany).

### Immunohistochemistry

Seven days after TONI, mice were deeply anesthetized with isoflurane, then proceeded with the cervical dislocation. Immunofluorescence staining was performed as described previously (Baldwin et al., 2015). Left eyes were fixed by placing the globe in 4% PFA overnight, dehydrated in 30% sucrose in PBS at 4°C (>24 h) and embedded in silicone molds containing optimal cutting temperature compound (OCT) (Sakura Finetek, Torrance, CA, United States). Eyes were then frozen on dry ice and sectioned at 10 μm through the dorsal–ventral/superior–inferior axis of the retina onto Superfrost Plus slides (VWR) using a Leica CM1860 cryostat (Leica, Wetzlar, Germany). Sections were simultaneously blocked and permeabilized by incubation in 5% normal goat serum for 60 min at room temperature. Sections were then incubated in primary antibody mouse anti-p-JNK (1:200, Cell Signaling Technology, Danvers, CO, United States) and rabbit anti-Iba-1 (1:400, Wako, Richmond, VA, United States) diluted in blocking solution overnight at 4°C. Sections were incubated in secondary antibodie DyLight 488, goat anti-rabbit IgG (Abbkine, San Diego, CA, United States) or DyLight 594, goat anti-mouse IgG (Abbkine, San Diego, CA, United States) diluted in blocking solution for 1 h at room temperature. Nuclei were counterstained with DAPI. Sections were imaged using a Leica DM6000 fluorescence microscope (Leica, Wetzlar, Germany). The p-JNK positive cells were analyzed using Image-Pro Plus (IPP) 6.0 software (MediaCybernetics, Bethesda, MD, United States) by a blinded investigator.

### Terminal-Deoxynucleotidyl Transferase Mediated Nick-End Labeling (TUNEL) Staining

To evaluate RGCs apoptosis, we performed transferase mediated nick-end labeling (TUNEL) staining in cross sections of retina. Retina sections producers were same as immunohistochemistry. TUNEL kit (Roche, Indianapolis, IN, United States) was used according to the manufacturer’s instructions. DAPI (Beyotime, Shanghai, China) was used for nuclear staining. Images were captured using a fluorescence microscope (Eclipse Ti-S; Nikon, Tokyo, Japan). The TUNEL-positive cells of the ganglion cell layer (GCL) were measured. The number of TUNEL-positive cells were determined in blinded fashion by an independent scientist. Image-pro plus (IPP) 6.0 software was used for immunofluorescence staining analysis.

### Western Blots

Left optic nerves were excised from the eye immediately under deep anesthesia with isoflurane and cervical dislocation, and the protein was extracted using a protein extraction kit (Beyotime Biotechnology, Shanghai, China). Equal amounts of protein sample were separated by SDS-polyacrylamide gel electrophoresis and then transferred to PVDF membranes (EMD Millipore, Inc., Billerica, MA, United States), which were blocked before being incubated with the appropriate primary antibodies overnight at 4°C. The following primary antibodies were used in the experiment: mouse anti-p-JNK (1:1000, Cell Signaling Technology, Danvers, CO, United States), mouse anti-JNK (1:1000, Cell Signaling Technology, Danvers, CO, United States), mouse-anti-Bcl2 (1:1000, Cell Signaling Technology, Danvers, CO, United States), rabbit anti-Bax (1:1000, Santa Cruz, CA, United States), mouse anti-iNOS (1:500, Abcam, Cambridge, MA, United States), and mouse anti-GAPDH (1:1000, Santa Cruz, CA, United States). Goat anti-rabbit IgG antibody conjugated with horseradish peroxidase (HRP) (1:2000, Abcam, Cambridge, MA, United States) or goat anti-mouse IgG antibody conjugated with HRP (1:2000, Cell Signaling Technology, Danvers, CO, United States) were selected for incubation with the membrane for 2 h at room temperature. Then, blot bands were visualized with an ECL reagent (Thermo Scientific, Pittsburgh, PA, United States) and photographed by a chemiluminescence imaging system (Bio-Rad, Hercules, CA, United States). The intensity of each band was quantified using Image Lab software (Bio-Rad, Hercules, CA, United States).

### Enzyme-Linked Immunosorbent Assay (ELISA)

The protein levels of TNF-α and IL-6 in the ipsilateral optic nerves were quantified by enzyme-linked immunosorbent assay (ELISA) according to the manufacturer’s instructions (Boster, Wuhan, China). The relative protein content is shown as nanograms per milligram of total protein.

### Statistical Analysis

Data are expressed as the mean ± standard deviation (SD). For normal distribution data, one-way analysis of variance (ANOVA) was used to compare the means of different groups. Two-way repeated measures ANOVA was used to analyze the F-VEP time course. The Bonferroni *post hoc* method was used to determine significant differences among groups. All statistical values were analyzed using SPSS 19.0 software (SPSS, Inc. Chicago, IL, United States). Statistical significance was accepted at *P* < 0.05.

## Results

### Time Course of F-VEP

**FIGURE 1:**
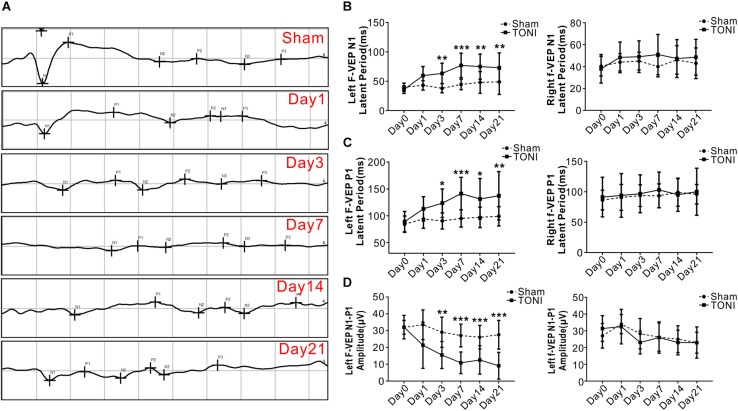
Latency and amplitude of F-VEP after TONI. The graph displays the mean and standard error bars of the F-VEP recordings. **(A)** Individual typical F-VEP recordings of each time point after TONI. **(B)** The left and right N1 latencies of each time point after Sham or TONI. **(C)** The left and right P1 latencies of each time point after Sham or TONI. **(D)** The left and right N1-P1 amplitudes of each time point after Sham or TONI. *N* = 10 per group, one-way ANOVA was used followed by Tukey’s HSD *post hoc* test and Holm–Bonferroni correction. ^∗^*P* < 0.05, ^∗∗^*P* < 0.01, ^∗∗∗^*P* < 0.001 vs. Sham group.

As shown in Figure 1, compared with those in the Sham mice, both the N1 and P1 latencies of left eyes were increased gradually in TONI mice from Day 1 and peaked on Day 7 (*P* < 0.001). The F-VEP reference of the dominant latency of the left N1-P1 wave (33.55 ± 8.79 μv) was established by examining Sham-operated mice. The left N1-P1 wave was significantly decreased from Day 1 after TONI, and reached a lowest level on Day 7 (10.85 ± 6.44 μv) (*P* < 0.001). There was no statistical difference of right N1 latency, P1 latency and N1-P1 amplitude of each time point.

### Time Course of Inflammatory and Apoptosis-Related Protein Levels in Optic Nerve After TONI

**FIGURE 2:**
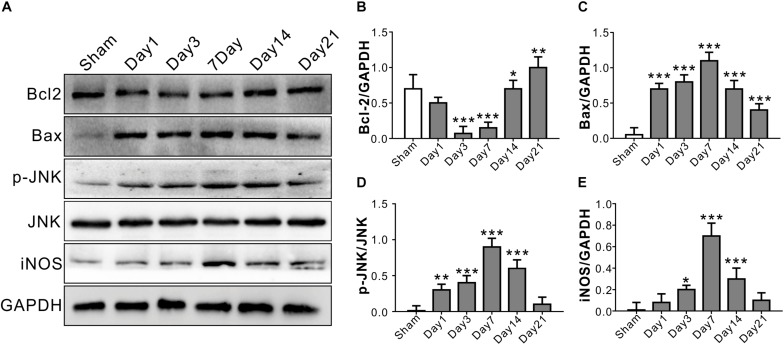
Time course of inflammatory and apoptosis-related protein levels in optic nerve after TONI. **(A)** Representative Western blot bands of Bcl2, Bax, p-JNK/JNK, and iNOS of each time point after injury. **(B–E)** Densitometric quantification Bcl2, Bax, p-JNK/JNK, and iNOS of each time point after injury. Data are represented as the mean ± SD. *N* = 5 per group, one-way ANOVA was used followed by Tukey’s HSD *post hoc* test and Holm–Bonferroni correction. ^∗^*P* < 0.05, ^∗∗^*P* < 0.01, ^∗∗∗^*P* < 0.001 vs. Sham group.

The anti-apoptotic protein Bcl2 level started decreasing from Day 1, and reached a relatively low level on Day 3 and Day 7 after TONI (*P* < 0.001). There were significant increases in Bax protein level over 21 days after TONI which peaked on Day 7 (*P* < 0.001). The expression of pro-inflammatory proteins p-JNK and iNOS were increased from Day 1, and peaked on Day 7 after TONI (Figure 2) (*P* < 0.001). Based on the time course of F-VEP and protein expression results, we choose Day 7 post-injury for the following study.

### COG1410 Attenuates Optic Nerve Apoptosis

**FIGURE 3:**
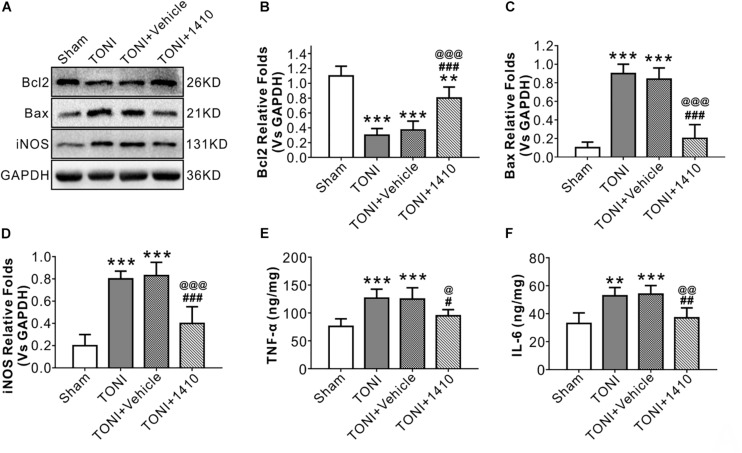
Effects of COG1410 on optic nerve inflammatory response and apoptosis-related protein expression. **(A–D)** Representative Western blot bands of densitometric quantification of Bcl2, Bax, and iNOS on Day 7 after injury. **(E,F)** Quantification of the pro-inflammatory factors TNF-α and IL-6 in the optic nerve by ELISA. Data are represented as the mean ± SD. *N* = 5 per group, one-way ANOVA was used followed by Tukey’s HSD *post hoc* test and Holm–Bonferroni correction. ^∗∗^*P* < 0.01, ^∗∗∗^*P* < 0.001 vs. Sham group; ^#^*P* < 0.01, ^##^*P* < 0.01, ^###^*P* < 0.001 vs. TONI group; ^@^*P* < 0.05, ^@@^*P* < 0.01, ^@@@^*P* < 0.001 vs. TONI + Vehicle group. Vehicle, sterile 0.9% of NaCl; 1410, COG1410.

Apoptosis-related protein levels were assessed by Western blotting. Apoptosis in the left optic nerve was confirmed by Bcl2 and Bax protein levels. Compared with those in the Sham group, the protein levels of Bcl2 in the TONI and TONI + vehicle groups were significantly decreased, and the protein levels of Bax in the TONI and TONI + vehicle groups were significantly increased (*P* < 0.001). However, COG1410 treatment significantly reversed these effects (Figures 3A–C) (*P* < 0.001).

### COG1410 Ameliorates the Inflammatory Response

**FIGURE 4:**
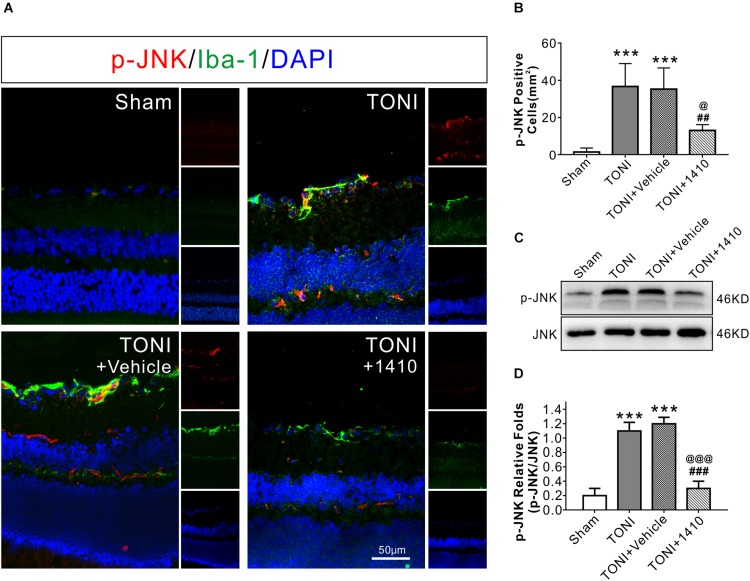
Effects of COG1410 on optic nerve and retinal p-JNK expression. **(A)** Spectral images showing representative immunofluorescent staining against p-JNK (red) and Iba-1 (green) in the retina on Day 7 after injury. **(B)** Quantification of p-JNK-positive cells by calculating the costained p-JNK and DAPI cells per mm^2^. **(C,D)** Representative Western blot bands of densitometric quantification of p-JNK/JNK on Day 7 after injury. Data are represented as the mean ± SD. *N* = 5 per group, one-way ANOVA was used followed by Tukey’s HSD *post hoc* test and Holm–Bonferroni correction. ^∗∗∗^*P* < 0.001 vs. Sham group; ^##^*P* < 0.01, ^###^*P* < 0.001 vs. TONI group; ^@^*P* < 0.05, ^@@^*P* < 0.01, ^@@@^*P* < 0.001 vs. TONI + Vehicle group. Scale bar = 50 μm. Vehicle, sterile 0.9% of NaCl; 1410, COG1410.

To determine the effects of COG1410 on inflammation, Western blotting, and immunofluorescence staining were carried out. The results showed that expression levels of the inflammation-related proteins iNOS (Figures 3A,D) and p-JNK (Figures 4C,D) in the ipsilateral optic nerve of TONI + COG1410 group were significantly decreased when compared with those in the TONI and TONI + vehicle group on Day 7 after injury (*P* < 0.001).

The relative protein concentrations of TNF-α and IL-6 were in the ipsilateral optic nerve measured to be 76.05 ± 13.56 and 33.08 ± 7.56 ng/mg, respectively, in the Sham group. The expression of TNF-α and IL-6 was significantly increased in the TONI group (126.76 ± 16.03, 52.76 ± 6.03 ng/mg) and TONI + vehicle group (125.04 ± 20.14, 54.04 ± 6.14 ng/mg) (*P* < 0.001). Compared with the TONI + vehicle group, COG1410 treatment significantly reduced the production of TNF-α (95.12 ± 11.03 ng/mg) (*P* < 0.05) and IL-6 (37.12 ± 7.02 ng/mg) (*P* < 0.01) (Figures 3E,F). Part of the p-JNK signals were co-localized with Iba-1 in the ipsilateral (left) GCL after TONI (Figure 4A). The fluorescence intensity of p-JNK was significantly increased in the TONI and TONI + vehicle groups compared with that in the Sham group (*P* < 0.001); however, COG1410 treatment significantly decreased p-JNK expression compared with the TONI + vehicle group (Figure 4B) (*P* < 0.05).

### Effects of COG1410 on RGC Apoptosis

**FIGURE 5:**
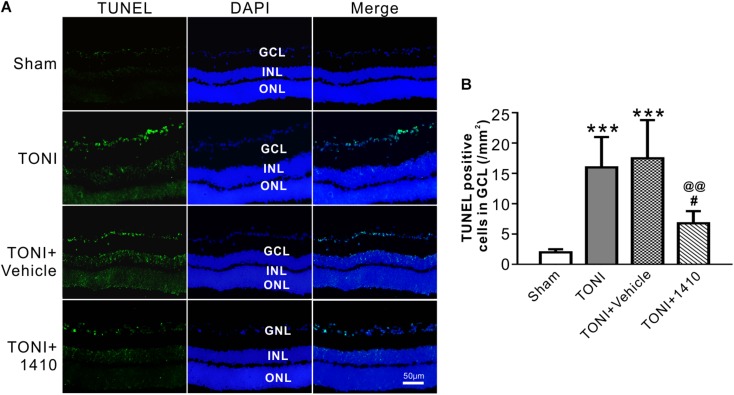
Effects of COG1410 on retinal apoptosis. **(A)** Spectral images showing the representative TUNEL staining of the retina on Day 7 after injury. **(B)** Quantification of TUNEL-positive cells in GCL. Data are represented as the mean ± SD. *N* = 5 per group, one-way ANOVA was used followed by Tukey’s HSD *post hoc* test and Holm–Bonferroni correction. ^∗∗∗^*P* < 0.001 vs. Sham group; ^#^*P* < 0.01, ^##^*P* < 0.01 vs. TONI group; ^@@^*P* < 0.01 vs. TONI + Vehicle group. Scale bar = 50 μm. Vehicle, sterile 0.9% of NaCl; 1410, COG1410; GCL, ganglion cell layer; INL, inner nuclear layer; ONL, outer nuclear layer.

Transferase mediated nick-end labeling assay was conducted to visualize cell apoptosis. The fluorescence detection of retinal cell apoptosis was performed on Day 7 after TONI. The ipsilateral retinal cell nuclei of the mouse in the Sham group were essentially negative for TUNEL staining. Compared with the Sham group, TUNEL-positive cells, predominantly in the GCL, were detected in the TONI and TONI + vehicle group. By contrast, compared with the TONI + vehicle group, the TUNEL-positive cells in the GCL were significantly decreased by 61.14% in the TONI + COG1410 group (Figure 5) (*P* < 0.01).

### Effects of COG1410 on Optic Nerve Edema and Visual Evoked Potentials

**FIGURE 6:**
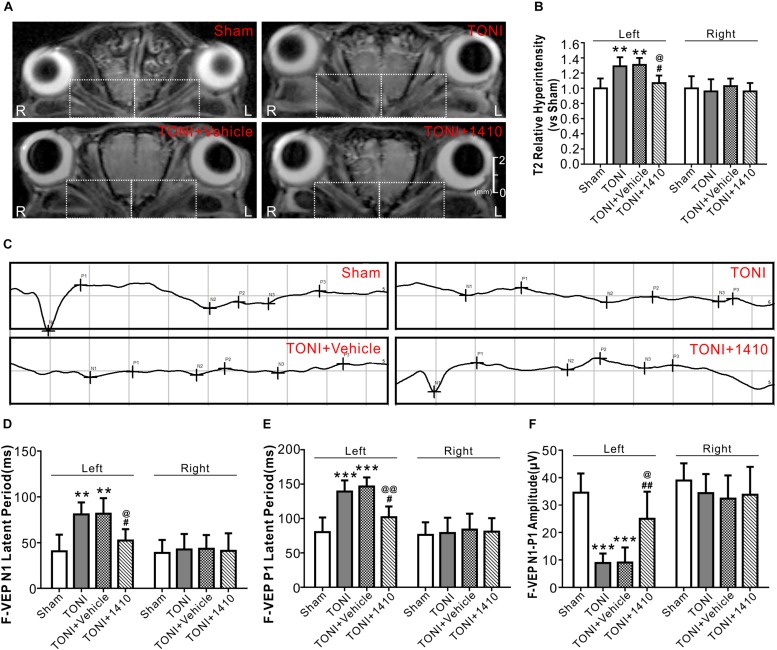
Effects of COG1410 on optic verve edema and visual function. **(A,B)** Representative images and quantification of relative T2 signal intensity values of ipsilateral optic nerve (left white dashed rectangle) and contralateral optic nerve (right white dashed rectangle) of T2WI on Day 7 after injury. *N* = 5 per group. **(C)** Representative VEP waves of mice on Day 7 after injury. **(D–F)** Statistics of the latency of N1, P1, and N1-P1 amplitude of each group. *N* = 10 per group. Data was represented as mean ± SD. One-way ANOVA was used followed by Tukey’s HSD *post hoc* test and Holm–Bonferroni correction. ^∗∗^*P* < 0.01, ^∗∗∗^*P* < 0.001 vs. Sham group; ^#^*P* < 0.01, ^##^*P* < 0.01 vs. TONI group; ^@^*P* < 0.05, ^@@^*P* < 0.01 vs. TONI + Vehicle group. R, right; L, left; Vehicle, sterile 0.9% of NaCl; 1410, COG1410.

T2WI MRI from a Bruker 7.0 T system was used to evaluate optic nerve integrity and edema. The representative T2 maps show the entire TONI lesions of each group of animals on Day 7 post-injury (Figure 6A). Compared with those in the Sham group, the left optic nerve T2 signal intensity values in the TONI and TONI + vehicle groups were significantly increased after injury (*P* < 0.01), whereas the signal was significantly decreased in COG1410-treated mice (Figure 6B, *P* < 0.05). There was no statistical difference of right optic nerve T2 signal intensity values of each group. These results indicate that COG1410 treatment improved optic nerve integrity and markedly reduced edema of the injured-optic nerve.

Traumatic optic nerve injury induction damaged the evoked potentials of ipsilateral vision (Figure 6C). Compared with the Sham group, the left N1 and P1 latencies were prolonged, and the left amplitude of N1-P1 was decreased in the TONI and TONI + vehicle groups. COG1410 treatment significantly reduced the left N1 latency (Figure 6D, *P* < 0.05) and P1 latency (Figure 6E, *P* < 0.01) but increased the left N1-P1 wave compared with the TONI + vehicle group (Figure 6F, *P* < 0.05). There was no statistical difference of right N1 latency, P1 latency and N1-P1 amplitude of each group.

## Discussion

In the current study, we investigated the anti-inflammatory, anti-apoptotic and RGC protective effects of COG1410 in a mouse model of TONI. Optic nerve inflammation and pro-inflammatory cytokine production were efficiently attenuated by COG1410 treatment. COG1410 alleviated optic nerve apoptosis characterized by modulation of apoptosis-related protein expression, decreased apoptotic rate of RGCs detected by TUNEL staining, reduced optic nerve edema and improved visual function as shown by F-VEP. These findings demonstrate the potential therapeutic properties of the apoE-mimic peptide COG1410 in TONI.

ApoE-deficient mice are cognitively impaired and exhibit more severe motor and cognitive deficits after closed head injury (Chen et al., 1997; Lynch et al., 2002). Previous studies have indicated that apoE plays an important role in optic nerve development and regeneration (Ignatius et al., 1986). Lopez-Sanchez et al. (2003) showed that apoE knockout mice have changes in optic nerve morphology and myelination. However, like most other proteins, the intact apoE holoprotein is too large to cross the blood–brain barrier (BBB) or blood-optic nerve barrier because of its 34-kDa molecular weight. Thus, from a translational perspective, the development of apoE-mimetic peptides with smaller molecular weights as alternative therapeutic choices for CNS disease treatment is greatly desirable.

COG1410 is a peptide derived from apoE amino acid residues 138–149 of the receptor region of the apoE holoprotein with aminoisobutyric acid (Aib) substitutions at positions 140 and 145 (acetyl-AS-Aib-LRKL-Aib-KRLL-amide). The therapeutic window for the treatment of acute brain injury animals expands to 2 h (Laskowitz et al., 2007), making it a potential protective agent for optic nerve injury treatment via intravenous administration. Several studies, including our previous research, assessed the neuroprotective effects of COG1410 on acute brain injury (Jiang and Brody, 2012; Li X. et al., 2018; Pang et al., 2018). COG1410 decreases JNK phosphorylation, iNOS protein levels and Bax expression, suggesting that COG1410 suppresses apoptosis possibly via inhibiting the p-JNK relative pathway. Furthermore, our radiological evidence indicated that treatment with COG1410 reduced the optic nerve edema and improved the optic nerve integrity.

**FIGURE 7:**
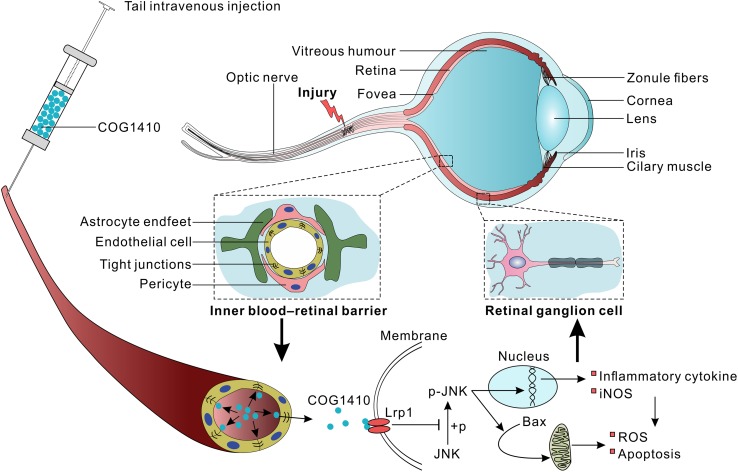
Proposed mechanism underlying the preservation of RGCs by COG1410 administration.

There are several possible interpretations of these results. As a mimic peptide of apoE, COG1410 might induce the release of the C-terminal fragment (CTF) of the apoE receptor low-density lipoprotein receptor-related protein-1 (LRP1) (Peng et al., 2019). CTF recruits JNK-interacting proteins (JIPs) and modulates JNK phosphorylation (Gotthardt et al., 2000). The results showed that the expression of p-JNK positive cells increased in GCL, which might be activated inflammatory microglia or Muller cells. The JNK signaling pathways are largely involved in the production of iNOS, modulating inflammatory mediators in activated macrophages/microglial cells and apoptosis (Pan et al., 2009; Manogaran et al., 2018; Papa et al., 2018). Therefore, we speculate that JNK is involved in the anti-inflammatory and anti-apoptotic effects of COG1410 (Figure 7).

Likewise, a certain number of RGCs that survive optic nerve damage is the foundation for optic nerve recovery after injury. Recovery of visual functions depends on the number of surviving RGCs at the early stage of injury. Our results show that administration of COG1410 significantly decreased p-JNK expression in the retina, reduced RGC apoptosis, and prevented the decrease in visual, as shown by F-VEP. Our recent study provides a link between the upregulation of p-JNK-mediated neuronal apoptosis in a stroke mouse model (Wu et al., 2016). Phosphorylated JNK contributes to RGC death, likely by cell death mechanisms (Welsbie et al., 2017; Liu C. et al., 2018). Together, these findings suggest that it may be useful in future studies to examine the ability of COG1410 to prevent RGC loss in the presence of TONI.

Several limitations need to be mentioned in this study. First, although it is still controversial, apoE polymorphisms are associated with outcomes of CNS diseases. Different alleles impart different functions (Bell et al., 2012). However, the effects of the apoE-mimic peptide COG1410 on TONI were tested only in one time point of WT mice in the current study. Gene knockout mice or transgenic mouse models in which the human APOE alleles have been “knocked in” at multiple days should be used in future studies. Second, additional studies are needed to examine whether targeted intravenous delivery is feasible in humans and leads to appropriate accumulation in the eye to establish clinical relevance. Other methods, such as intranasal administration, have become a common delivery method for the treatment of CNS disease (Kumar et al., 2018). Intranasal administration might allow for more frequent dosing, reduced patient discomfort and reduced risk of complications compared with other medication delivery routes. It will be important to complete carefully controlled studies to confirm the therapeutic effect of COG1410 by intranasal administration. We are currently undertaking these studies.

## Conclusion

The present study demonstrates that COG1410 alleviates vision loss after TONI by suppressing inflammation, edema and apoptosis, which reduces RGC loss. Our current findings suggest that COG1410 is a promising preclinical therapeutic agent for the treatment of TONI.

## Data Availability

The raw data supporting the conclusions of this manuscript will be made available by the authors, without undue reservation, to any qualified researcher.

## Ethics Statement

We followed the Guide for the Care and Use of Laboratory Animals of China, the number of mice was minimized as shown in the figures, and all the experimental mice were euthanized under deep anesthesia.

## Author Contributions

XZ and LK designed the study. LK and JP contributed to the sample preparation, data collection and analysis, and wrote the manuscript. XZ, YJ, and ZZ directed the experiments. All authors reviewed the manuscript.

## Conflict of Interest Statement

The authors declare that the research was conducted in the absence of any commercial or financial relationships that could be construed as a potential conflict of interest.
